# Trends in Fatal Poisoning Among Drug Users in France From 2011 to 2021: An Analysis of the DRAMES Register

**DOI:** 10.1001/jamanetworkopen.2023.31398

**Published:** 2023-08-30

**Authors:** Bruno Revol, Théo Willeman, Marc Manceau, Véronique Dumestre-Toulet, Jean-Michel Gaulier, Nathalie Fouilhé Sam-Laï, Hélène Eysseric-Guérin

**Affiliations:** 1 Addictovigilance Department, Grenoble Alpes University Hospital, Grenoble, France; 2 HP2 Laboratory, Inserm U1300, Grenoble Alpes University, Grenoble, France; 3 Laboratory of Pharmacology, Pharmacogenetics, and Toxicology, Grenoble Alpes University Hospital, Grenoble, France; 4 Clinical Forensic Medicine Department, Grenoble Alpes University Hospital, Grenoble, France; 5 Clinical Research Center, Inserm CIC1406, Grenoble Alpes University Hospital, Grenoble, France; 6 ToxGen Laboratory, Bordeaux, France; 7 Toxicology Unit, Lille University Hospital, Lille, France; 8 Forensic Laboratory, Grenoble Alpes University, Grenoble, France

## Abstract

**Importance:**

The DRAMES (Décès en Relation avec l’Abus de Médicaments Et de Substances) register is a database of drug-related deaths with the aim of identifying the psychoactive substances associated with and estimating the trends in these deaths. Our novel approach is based on the collection of data on all deaths for which toxicology experts have performed analyses.

**Objective:**

To describe drug-related deaths in France and report trends over an 11-year period.

**Design, Setting, and Participants:**

This case series used a national register to assess 4460 drug-related deaths that occurred from 2011 to 2021 in France. Data analyses were performed from January 1, 2012, to December 31, 2022.

**Main Outcomes and Measures:**

Demographic characteristics; medical and substance abuse history; forensic autopsy findings; and toxicology reports.

**Results:**

Among the 4460 deceased individuals (mean [SD] age, 37.8 [10.5] years), the mortality rate was highest among men (sex ratio, 4.4:1). Of the deaths involving a single or predominant drug, the legal substitution product, methadone, was the leading cause of death during the entire study period, ahead of heroin—44.7% and 35.9% for methadone vs 15.8% and 21.8% for heroin in 2011 and 2021, respectively. Between 2011 and 2021, most of the drug-related deaths shifted from licit to illicit drugs, and statistically significant variations were found for buprenorphine, cocaine, heroin, methadone, and other licit opioids. Deaths related to polydrug use increased from 23.2% in 2011 to 30.6% in 2021. In this context, opioids remained associated with most deaths, with at least 1 opioid being involved in approximately 9 of 10 cases (85.9%) in 2021. However, the main trend was the dramatic increase in drug combinations with cocaine, from less than one-third of cases in 2011 (30.8%) to more than half in 2021 (57.8%).

**Conclusions and Relevance:**

This case series assessment of 4460 drug-related deaths found that opioids used alone or in combination were the main contributor to drug-related deaths, despite having a lower prevalence than other drugs. This finding is similar to that of other countries; however, in France licit methadone was the leading cause of opioid-related deaths (ahead of heroin) during the study period. Deaths associated with use of cannabis, new psychoactive substances, and stimulants (including amphetamine-type stimulants and cocaine, especially in combination) have increased and should be closely monitored.

## Key Points

**Question:**

Which psychoactive substances (medications or illicit drugs) were involved in deaths in France during the period from 2011 to 2021?

**Findings:**

This case series assessment of 4460 drug-related deaths in France found that opioid drugs were the main contributor to deaths, as they are in most countries. However, licit methadone was the main cause of opioid-related deaths during the study period, with 36% of deaths involving a single or predominant drug and 42% of deaths involving drug combinations in 2021.

**Meaning:**

The findings of this case series indicate that a large-scale naloxone distribution program is urgently needed in France to prevent opioid overdoses, including among licit methadone users.

## Teaser

This cases series includes all drug-related deaths with available toxicology reports for an 11-year period in France and assesses the trends in psychoactive substance use.

## Introduction

According to the recent *World Drug Report* from the United Nations Office on Drugs and Crime (UNODC), an estimated 494 000 people worldwide died of drug use in 2019, and there has been an overall increase in drug-related deaths of 17.5% between 2009 and 2019.^1^ However, there is considerable regional variation. Although the US reported age-adjusted mortality associated with drug overdose to be 216.0 per million among the population 15 to 64 years of age in 2019, the European Union (EU) reported only 14.8 deaths per million for the same age bracket in the same year. Thus, it is estimated that 5141 deaths from overdose involving illicit drugs occurred in the EU in 2019, with a notable increase in most of the Southern and Eastern EU countries when compared with 2018. More than three-quarters (77%) occurred among men and among 35- to 39-year-olds, with a mortality rate of 37.7 deaths per million, more than double the average seen for all other age bands.^2^

In France, the DRAMES (Décès en Relation avec l’Abus de Médicaments Et de Substances) register is a database of drug-related deaths that aims to identify the psychoactive substances involved (medications or illicit drugs), assess their dangerousness, and estimate the trends in the number of these deaths. The register is based on the prospective annual nationwide collection of data on all deaths for which toxicology analyses have been performed. All drug-related deaths are notified to France’s National Agency for the Safety of Medicines and Health Products. Postmortem toxicologic analyses are performed as part of the search for the cause of death, but only at the request of the judicial authorities. When there is no suspicion of drug implication in a death, an analysis is not usually ordered. Reporting was voluntary. The Grenoble Addictovigilance Centre (coordinator of this study since 2011) evaluated each case to determine the drug or drugs involved in the death. The purpose of this report was to present the trends in the drugs and classes of drugs implicated in drug-related deaths during 11 years from 2011 to 2021.

## Methods

Because this report used only routinely collected, deidentified data, it did not require any ethics committee approval, and informed consent was waived, in accordance with French regulations for mandatory reporting by health care professionals.^3^ We followed the reporting guideline for case series, which requires explicit discussion of eligibility criteria; appropriate statistics with assumptions that are appropriate for the setting (Bonferroni correction); and the study limitations as well as how they could be overcome.

### Data Collection

Data were collected from the forms completed by the toxicologist that included the following information for each individual: demographic characteristics; medical and substance abuse history; circumstances in which the body was found; autopsy and pathological findings; identification of the substances found in analyses of biological specimens and quantities in the blood or in other matrices if blood specimen was not available; and probable cause of death. These data made it possible to classify each case as either directly substance-related cause of death (toxic cause alone, usually overdose; and toxic cause with related pathology) or indirectly substance-related (eg, falling from a high place while under the influence of cocaine). Indirectly drug-related deaths were not included in this report.

### Inclusion and Exclusion Criteria

The DRAMES register includes deaths that meet the European Monitoring Centre for Drugs and Drug Addiction definition of *drug-related deaths*.^4^ Specifically, cases were included when the underlying cause of death was drug-induced psychosis, drug dependence, nondependent drug abuse, accidental or self-inflicted poisoning, and poisoning with undetermined intent. Cases were included when the death was associated with a standard list of specific drugs or drug classes: cannabis, cocaine, hallucinogens, opioids, stimulants, or abuse of any other psychoactive substance assessed as being responsible for the death. The term “X-related death” means that X was the single or predominant drug that caused the death, or that X was 1 of the drugs that caused death.

The following DRAMES records were excluded from the study sample: deaths by suicide (eg, when the suicide was addressed in writing and/or the means was by hanging); deaths caused by a third party (homicide); deaths associated with accidental intoxication in children (excluding drug dependence); deaths due solely to intoxication with licit and prescribed drugs, without a documented history of substance abuse (eg, death associated with antidepressants); cases in which another cause, not related to a psychoactive substance, was found; cases with insufficient documentation (eg, no known cause of death); cases without drug testing (in the absence of blood, analyses of other matrices, eg, bile or muscle); deaths associated with vehicular crashes (drivers or passengers).

### Imputability (Death Associated With Substance Abuse)

For each case evaluated, each substance present in the blood was subject to an imputability analysis regarding the death, scored from high (level 1) to low (level 4). To differentiate the substances identified in the blood from the substance(s) involved in the death, therapeutic, toxic, and lethal blood concentrations^5,6,7^ were used to grade substance abuse as follows: lethal concentration = level 1; toxic concentration = level 1 or 2 depending on other substances present; therapeutic concentration = level 1, 2, 3 or 4 depending on other substances present; and subtherapeutic concentration = no score assigned. For this study, only level 1 was considered to be a cause of death. Depending on the number of substances involved, the score was subclassified as 1.0, meaning that only 1 drug was present (eg, methadone without any other substance); 1.1, meaning 1 predominant drug (including active metabolites; eg, methadone at a toxic concentration combined with benzodiazepine at a therapeutic concentration); 1.2 meaning 2 codominant drugs were present (eg, methadone at a toxic concentration combined with benzodiazepine at a lethal concentration); and 1.3 meaning 3 or more codominant drugs were present (eg, methadone combined with heroin and cocaine).

To distinguish heroin-related deaths from morphine-related deaths, apart from heroin itself, 1 or more of the following opium metabolites or alkaloids had to be present: 6-monoacetylmorphine, papaverine, or noscapine. According to the grading scheme, several drugs could be considered as the cause of death. The imputability of the drug(s) in each case was independently assessed by 2 toxicology experts (N.F.S.-L. and H.E.G.). In case of disagreement, the advice of a third expert (B.R. or T.W.) was sought.

### Statistical Analysis

We performed χ^2^ tests of homogeneity for selected drugs or drug classes over the whole study period. All analyses were conducted with R, version 3.6.3 (R Foundation for Statistical Computing), and its tidyverse meta-package, including the ggplot2 package for plotting. Smoothed curves correspond to LOESS (locally estimated scatterplot smoothing) curves obtained using ggplot2. To account for the multiplicity of comparisons for homogeneity tests, the statistical significance threshold for *P* values was adapted using a Bonferroni correction (*P* < .0025). Data analyses were performed from January 1, 2012, to December 31, 2022.

## Results

**Figure 1.**
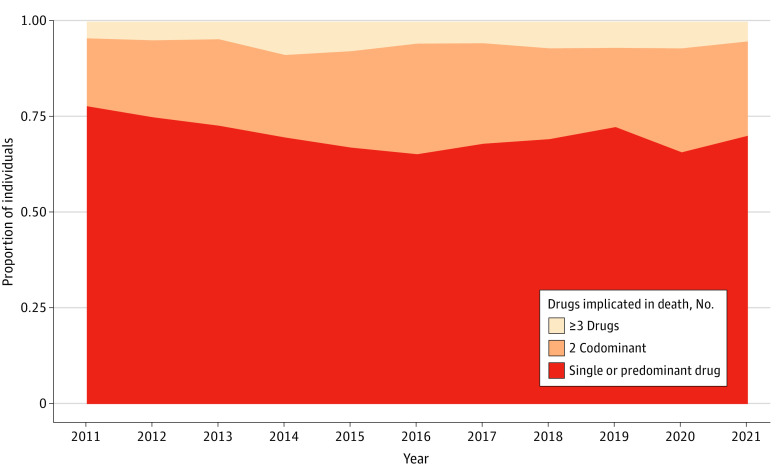
Trends in Drug-Related Deaths Involving a Single Drug, a Predominant Drug, 2 Codominant Drugs, or 3 or More Codominant Drugs, France, 2011 to 2021

In all, 4460 cases of individuals (mean [SD] age, 37.8 [10.5] years; 3623 [81.2%] men, 831 [18.6%] women, and 6 [0.1%] of unknown sex) who died of drug-related causes from January 1, 2011, to December 31, 2021, were included. The overdose mortality rate was highest among men (sex ratio, 4.4:1), with a progressive increase in the age of the affected population over time. The demographic characteristics of the study population are presented by year in the Table. During the survey period, the participation of toxicology experts increased, reaching 47 experts in 27 public and private accredited (International Organization for Standardization, 15189 or 17025) forensic laboratories throughout France, corresponding to a coverage of 81% of the national territory in 2020. Deaths related to polydrug use increased during the period from 23.2% in 2011 to 30.6% in 2021 (Figure 1).

**Table.** Demographic Characteristics and Medical and Substance Abuse History of Included Individuals and Number of French Departments^a^ Covered, by Year

Characteristic	2011	2012	2013	2014	2015	2016	2017	2018	2019	2020	2021	Total
Participants, No. (%)	280	310	285	243	343	406	432	464	503	567	627	4460
Age, mean (SD), y	34.9 (10.1)	35.6 (9.77)	35.2 (9.75)	36.7 (9.72)	36.1 (10.2)	38.2 (9.81)	38.9 (10.3)	38.0 (10.9)	39.1 (10.8)	38.4 (10.4)	39.4 (11.0)	37.8 (10.5)
Sex ratio (male:female)	4.2	4.3	4.5	4.9	4.4	5.7	4.9	4.9	3.5	3.7	4.3	4.4
Departments covered, No. (%)	58 (57.4)	69 (68.3)	69 (68.3)	64 (63.4)	69 (68.3)	77 (76.2)	71 (70.3)	81 (80.2)	77 (76.2)	82 (81.2)	78 (77.2)	NA
Drugs involved,^b^ No.												
Single (1.0) /predominant drug (1.1)	215 (76.8)	231 (74.5)	206 (72.3)	168 (69.1)	226 (65.9)	262 (64.5)	290 (67.1)	318 (68.5)	360 (71.6)	368 (64.9)	435 (69.4)	3079 (69.0)
2 Codominant drugs (1.2)	49 (17.5)	62 (20)	64 (22.5)	52 (21.4)	85 (24.8)	116 (28.6)	112 (25.9)	109 (23.5)	103 (20.5)	152 (26.8)	153 (24.4)	1057 (23.7)
≥3 Codominant drugs (1.3)	16 (5.7)	17 (5.5)	15 (5.3)	23 (9.5)	32 (9.3)	28 (6.9)	30 (6.9)	37 (8.0)	40 (8.0)	47 (8.3)	39 (6.2)	324 (7.3)
**Place of death, No. (%)**												
Home	189 (67.5)	201 (64.8)	218 (76.5)	167 (68.7)	257 (74.9)	290 (71.4)	302 (69.9)	328 (70.7)	376 (74.8)	402 (70.9)	476 (75.9)	3206 (71.9)
Public road	12 (4.3)	21 (6.8)	12 (4.2)	15 (6.2)	13 (3.8)	23 (5.7)	30 (6.9)	25 (5.4)	33 (6.6)	39 (6.9)	47 (7.5)	270 (6.1)
Hospital	15 (5.4)	13 (4.2)	9 (3.2)	14 (5.8)	22 (6.4)	22 (5.4)	31 (7.2)	33 (7.1)	31 (6.2)	55 (9.7)	31 (4.9)	276 (6.2)
Prison	14 (5.0)	12 (3.9)	13 (4.6)	4 (1.6)	6 (1.7)	10 (2.5)	3 (0.7)	9 (1.9)	9 (1.8)	11 (1.9)	8 (1.3)	99 (2.2)
Party environment	3 (1.1)	6 (1.9)	2 (0.7)	2 (0.8)	8 (2.3)	3 (0.7)	3 (0.7)	7 (1.5)	4 (0.8)	1 (0.2)	6 (1.0)	45 (1.0)
Other/unknown	47 (16.8)	57 (18.4)	31 (10.9)	41 (16.9)	37 (10.8)	58 (14.3)	63 (14.6)	62 (13.4)	50 (9.9)	59 (10.4)	59 (9.4)	564 (12.6)
**History of substance abuse, No. (%)**												
Drug abuse/dependence	150 (53.6)	164 (52.9)	177 (62.1)	130 (53.5)	178 (51.9)	220 (54.2)	213 (49.3)	198 (42.7)	186 (37.0)	235 (41.4)	285 (45.5)	2136 (47.9)
Alcoholism	64 (22.9)	66 (21.3)	87 (30.5)	49 (20.2)	71 (20.7)	96 (23.6)	109 (25.2)	120 (25.9)	141 (28.0)	139 (24.5)	132 (21.1)	1074 (24.1)
Ongoing opioid substitution therapy	67 (23.9)	52 (16.8)	57 (20.0)	43 (17.7)	57 (16.6)	77 (19.0)	73 (16.9)	70 (15.1)	86 (17.1)	100 (17.6)	111 (17.7)	793 (17.8)

Abbreviation: NA, not applicable.

^a^ France comprises 101 administrative departments.

^b^ Only level 1 (lethal) concentration of a drug was considered to be the cause of death. Scoring added a subclassification for the number of substances present: 1 drug alone (eg, methadone at lethal concentration), score = 1.0; 1 predominant drug (eg, methadone at toxic concentration with benzodiazepine at therapeutic concentration), score = 1.1; 2 codominant drugs (eg, methadone at toxic concentration with benzodiazepine at lethal concentration) = 1.2; and ≥3 or more codominant drugs (eg, methadone with heroin and cocaine), score = 1.3.

**Figure 2.**
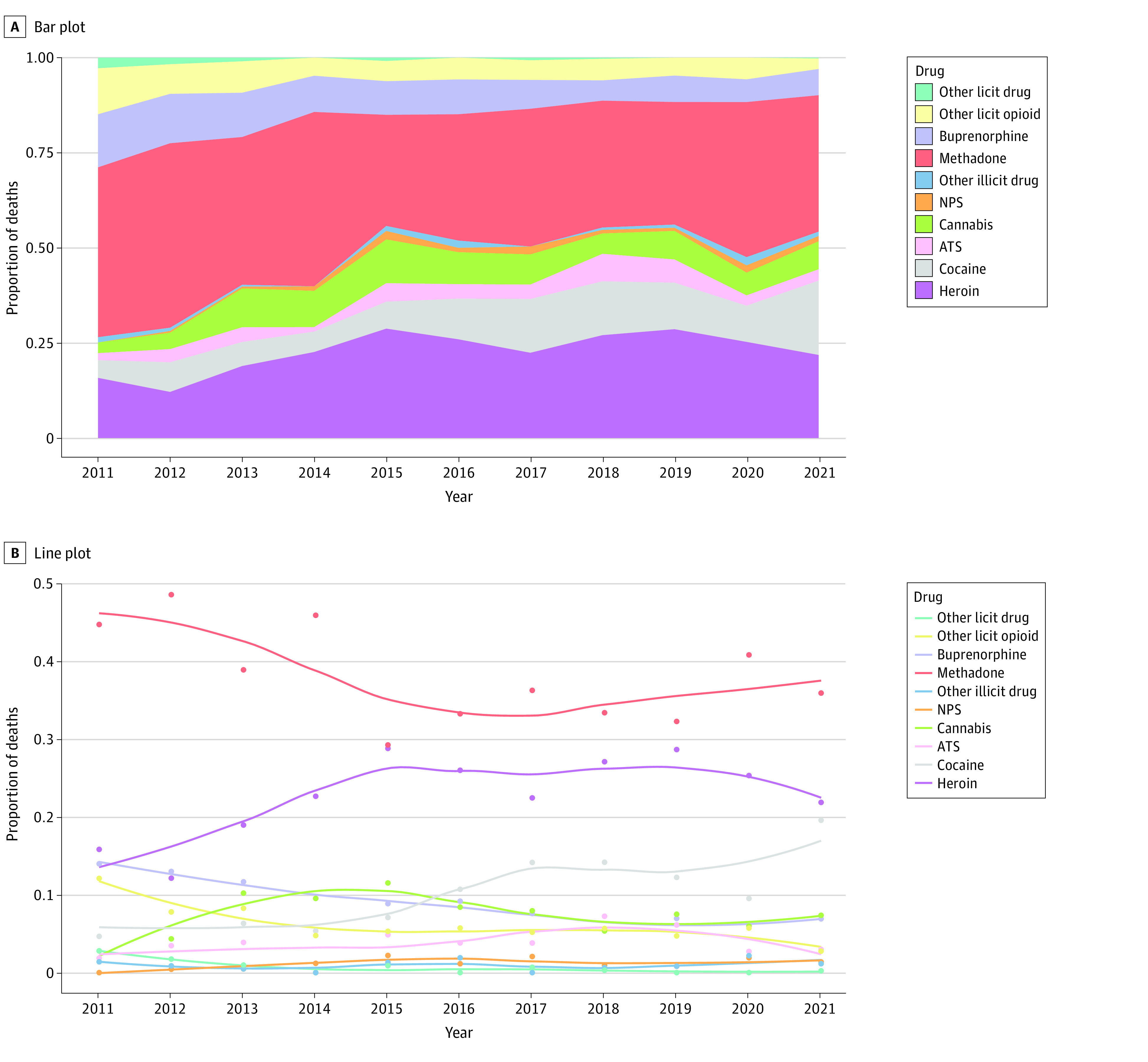
Trends in Drug-Related Deaths Involving a Single Drug or a Predominant Drug, by Class of Drug, France, 2011 to 2021 ATSs refers to amphetamine-type stimulants and included amphetamine, DOC, MDMA, and methamphetamine; NPSs refers to new psychoactive substances and included carfentanil, ethylphenidate, MDPHP, MDPV, 3- and 4-MMC, MXE, methylone, ocfentanil, U-47700, 25I-NBOMe, 4-CMC, 4-MEC, 5-MAPB; other illicit drugs included butane, GBL, GHB, ketamine, LSD, mitragynine, nitrous oxide, and poppers; other licit drugs included alprazolam, clobazam, clonazepam, cyamemazine, ether, hydroxyzine, mephenesin, meprobamate, nefopam, oxazepam, phenobarbital, pregabalin, propofol, and zolpidem; and other licit opioids included alfentanil, codeine, dihydrocodeine, fentanyl, morphine, oxycodone, pholcodine, remifentanil, and tramadol.

The trends in drug-related deaths involving a single drug (1.0) or 1 predominant drug (1.1) are presented in Figure 2. Over the whole period, most deaths shifted from licit (buprenorphine, licit opioids, methadone, and others) to illicit drugs (amphetamine-type stimulants [ATSs], cannabis, cocaine, heroin, new psychoactive substances [NPSs], and others) (Figure 2A). The relative incidence of heroin-related deaths increased from 15.8% in 2011 to a maximum of 28.8% in 2015. The incidence of cocaine-related deaths increased from 4.7% in 2011 to a maximum of 19.5% in 2021. The incidence of ATS-related deaths increased from 1.9% in 2011 to a maximum of 7.2% in 2018. The incidence of cannabis-related deaths increased from 2.8% in 2011 to a maximum of 11.5% in 2015; and the incidence of NPS-related deaths increased to 2.2% in 2015. Methadone, a legal substitution product, remained the leading cause of death over the entire period, ahead of heroin, reaching respectively 35.9% and 21.8% in 2021. Interestingly, the curves for methadone and heroin mirror each other over the entire period (Figure 2B). The incidence of buprenorphine-related deaths and other licit opioid-related deaths decreased from 14.0% and 12.1% in 2011 to 6.9% and 2.8% in 2021, respectively. Over the period, statistically significant variations were found for buprenorphine (*P* = .002), cocaine (*P* < .001), heroin (*P* < .001), methadone (*P* < .001), and other licit opioids (*P* = .002) (eTable 1 in Supplement 1).

**Figure 3.**
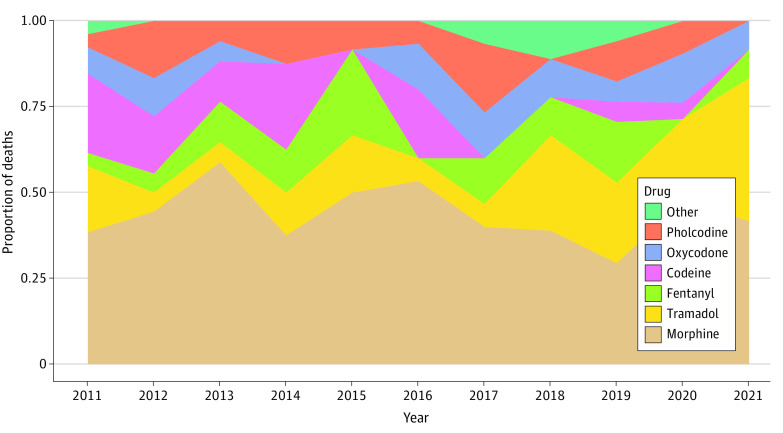
Trends in Licit Opioid-Related Deaths Involving a Single Drug or a Predominant Drug, Excluding Methadone and Buprenorphine, by Class of Drug, France, 2011 to 2021 Other included alfentanil, dihydrocodeine, and remifentanil.

Among licit opioid-related deaths involving a single drug (1.0) or a predominant drug (1.1) (Figure 3), morphine was most frequently implicated in deaths throughout the whole period, but tramadol-related deaths have been increasing since 2017. In contrast, the number of codeine-related deaths decreased. Over the 11-year period, deaths attributed to fentanyl appear to be replaced by death associated with oxycodone and vice versa, with no significant difference when these 2 strong opioids are considered together.

**Figure 4.**
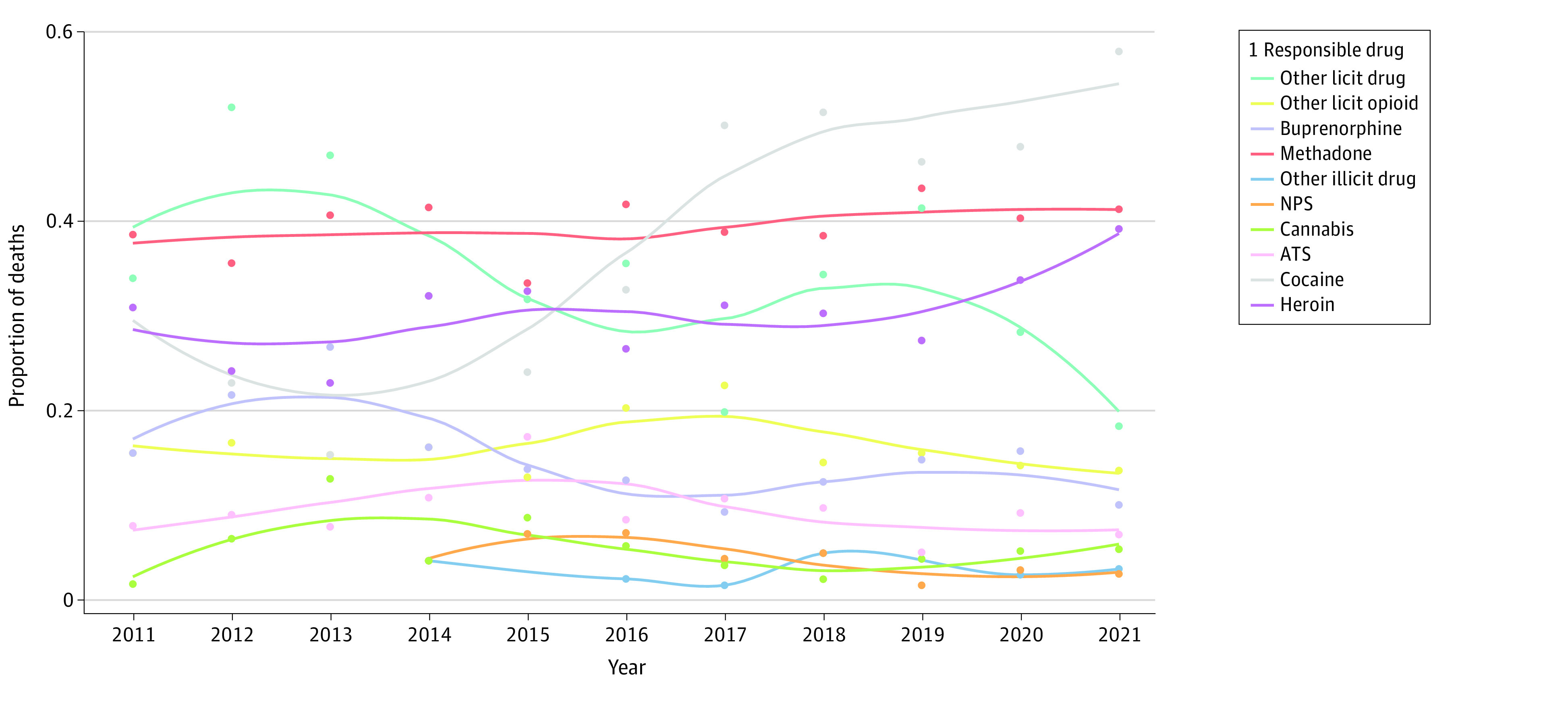
Trends in Drug-Related Deaths Involving 2 or More Codominant Drugs, by Class of Drug, France, 2011 to 2021 ATSs refers to amphetamine-type stimulants and included amphetamine, MDMA, methamphetamine, PMMA; NPSs included alpha-PVP, butylone, deschloroetizolam, diclazepam, ethylphenidate, MDPV, 3- and 4-MMC, MPA, MXP, ocfentanil, 3-FPHEN, 3-MeO-PCP, 4-FMA, 4-FMPH, 4-MEC, 4-MPD, 5-APB, 5-APDB, and 5-MeO-DALT; other illicit included GHB, ketamine, and poppers; other licit drugs included alimemazine, alprazolam, amisulpride, amitriptyline, baclofen, bromazepam, citalopram, clobazam, clonazepam, clomipramine, clozapine, cyamemazine, diazepam, diphenhydramine, doxylamine, duloxetine, fluoxetine, gabapentin, hydroxyzine, levomepromazine, lidocaine, loxapine, maprotiline, meprobamate, methylphenidate, mianserin, mirtazapine, nordiazepam, olanzapine, oxazepam, paroxetine, phenobarbital, pregabalin, promethazine, propranolol, phenytoin, quetiapine, sertraline, tiapride, tropatepine, valproic acid, venlafaxine, zolpidem, zopiclone, zuclopenthixol; other licit opioids included codeine, dextromethorphan, fentanyl, morphine, oxycodone, pholcodine, and tramadol.

Lastly, Figure 4 shows drug-related deaths associated with 2 or more codominant drugs (1.2 and 1.3, respectively). In the context of polydrug use, opioids remained associated with the greatest number of deaths in 2021, with at least 1 opioid involved in approximately 9 of 10 cases (85.9%). More specifically, 41.1% of combinations included methadone; 39.1%, heroin; 9.9%, buprenorphine; and 13.5%, a licit opioid other than methadone or buprenorphine (Figure 4). There was a dramatic increase in combinations involving cocaine, from less than one-third of cases in 2011 (30.8%) to more than half in 2021 (57.8%). In contrast, the relative increases in numbers of deaths related to combinations with cannabis, ATSs, or NPSs observed in 2013 (12.7%), 2015 (17.1%) and 2016 (6.9%), respectively, have not been confirmed since that time. In 2021, a licit nonopioid drug (primarily a hypnotic, anxiolytic, antidepressant, or antipsychotic) was associated with fewer than 1 in 5 cases. The main substances responsible or coresponsible for death are shown in the eFigure in Supplement 1.

## Discussion

As elsewhere in Europe, the death rate associated with drug abuse in France was highest among men from 35 to 39 years of age.^2^ Our survey covering the period from 2011 to 2021 shows an increase in polydrug use resulting in fatal overdoses in France. Worldwide, polydrug use is relatively infrequent among the general population, varying between 0.3% in Portugal to 3.4% in Uruguay.^8^ However, polydrug use is far more common among people engaging in high-risk drug practices.^9^ For example, of 1311 used syringes collected from 8 European cities in 2019, 32% contained multiple drugs.^10^

In France, as in most countries, opioids are by far the drug class most frequently associated with drug-related mortality. Globally, in 2020, 64% of all direct drug-related deaths reported to the UNODC concerned opioids.^1^ In the EU, opioids (including heroin and its metabolites) often in combination with other substances, were present in three-quarters (74%) of fatal overdoses reported in 2020.^11^ Opioids other than heroin, including buprenorphine, fentanyl and its derivatives, methadone, or tramadol were associated with a substantial share of overdose deaths in some European countries.^2^ For example, an increase in direct drug-related mortality in 2020 was reported in Belarus, partly related to the presence of illicitly manufactured methadone available on the black market.^2^

The French situation seems different since 2010^12^ because licit methadone has been the main cause of opioid-related deaths for many years, ahead of heroin, although the distribution of licit drug-related deaths has been decreasing during the period. This trend is not only true for opioid-related deaths involving a single drug or a predominant drug, but methadone was also the leading opioid used in combination with other drugs during the study period.

In contrast, our study confirms the relative safety of buprenorphine in terms of death from overdose (compared with methadone) in France^13^ or in England and Wales.^14^ It is estimated that 1 in 2 high-risk opioid users receives opioid substitution treatment (ie, buprenorphine and methadone) in the EU, but large variations exists between countries, ranging from 10% in Latvia to more than 80% in France.^12^ Opioid replacement therapies have repeatedly been shown, across cultures, settings, and time periods to substantially reduce mortality among heroin users entering treatment. However, methadone was the principal drug involved in fatal poisonings in Denmark during 2007 to 2017^15^ and was implicated in almost as many drug-related deaths as heroin in the UK in the 1990s.^16^ Subsequently, the number of methadone-related deaths was reduced by a factor of 4 after the introduction of supervised methadone use in Scotland and England.^17^ In 2005, in England, supervised consumption was applied to 35.7% of all methadone prescriptions^18^ because of the introduction of specific funding for community pharmacists. In France, no such practice has yet been established, although the supervision of consumption is recommended at the start of treatment.

The COVID-19 pandemic influenced the availability of both illicit and licit drugs. In France, an increase in methadone-related deaths (1.0 or 1.1) was observed in 2020. As in several countries, opioid substitution treatment services exercised more relaxed supervision. This flexibility allowed patients to temporarily take home up to 28 days of methadone doses, which may explain the relative increase in methadone-related deaths in 2020. However, the increase in take-home methadone doses was not associated with negative treatment outcomes (including overdose events) in the US.^19^ Another explanation could be the lower availability of illicit drugs during lockdowns. Localized shortages of heroin were indeed reported in some EU countries in the early stages of the pandemic and may have contributed to an increase in the use of replacement substances.^20^ This is consistent with an increase in buprenorphine-related deaths at the time of COVID-19 restrictions reported in Finland,^21^ and with the increase in buprenorphine and methadone misuse reported in France during this period.^22^

Semisynthetic or synthetic opioid analgesics are a particular concern because of the central role this group plays in the opioid epidemics in Canada and the US.^23^ However, no significant increase in fentanyl- or oxycodone-related deaths was observed in France during 2011 to 2021. Neither the frequent off-label use (oral transmucosal fentanyl formulations prescribed for noncancer pain) and the risk of primary fentanyl dependence^24^ nor the large increase in the number of individuals with “doctor-shopping” behavior for oxycodone (a proxy for potential misuse or abuse)^25^ resulted in a significant increase in mortality. In light of the US crisis, the French harm-reduction model, the legal framework for access to strong opioids, and the multimodal approach to pain treatment may prove inspiring.^26^ In contrast, our data suggest an increase in tramadol-related deaths, which is consistent with an increase in illegal ways of procurement, high-risk use driven by its psychoactive effects sought to control withdrawal symptoms, and self-medication for anxiety or depressive symptoms.^27^

Worldwide, few countries (11%) reported stimulant drugs as causing a large number of drug-related deaths.^1^ One exception was Mexico, where amphetamines were reported to be the most frequently detected drugs among those who died of a drug overdose.^1^ The second leading cause of drug-related death (after opioids) in France were stimulant drugs, including cocaine, ATSs, cathinones, and some other NPSs. Our observations are in line with a previous French study that reported an increase in cocaine-related deaths since 2014.^28^ This very important trend has been confirmed elsewhere in Europe, notably in the UK with an increase in cocaine-related deaths that began in 2010 and accounted for one-quarter of drug-related deaths in 2019.^1^ According to our study findings, cocaine is now involved in more than half of polydrug-induced deaths reported in France.

Of the stimulant drugs, ATSs are second to cocaine in terms of deaths in France. Among 20 countries with postmortem data available for 2020, Austria, Czechia, Estonia, Finland, Norway, and Slovakia reported an increase in the number of deaths involving amphetamines compared with the previous year.^11^ This trend was not observed in France; however, the disruption in the nightlife economy during the COVID-19 pandemic may well have affected trends in stimulant use. Preliminary data for 2021 suggest a return to prepandemic levels for cocaine use in Europe.^11^

Finally, analysis of used syringes collected in Paris in 2018 showed that 51% contained cathinones, a proportion that increased to 67% in 2019.^10^ Although these results do not translate into a significant increase in NPS-related deaths, careful monitoring is necessary; for example, in the Netherlands, the number of poisonings suspected to involve 3-methylmethcathinone increased from 10 in 2018 to 64 in 2020.^11^

The only other substance identified as the sole cause of direct drug-related deaths in our analysis was cannabis. Cannabis remains by far the most widely used illicit drug both in France and worldwide. It should be noted that cannabis is frequently implicated in traffic fatalities,^29^ but these incidents are excluded from the DRAMES register. Globally, in 2020, 4% of all drug-related deaths reported to the UNODC were related to cannabis.^1^ In France, since 2006, cannabis-related cardiovascular complications and deaths have been reported among young and apparently healthy cannabis users.^30,31^ According to our results, cannabis is probably an underestimated cause of direct drug-related deaths, despite its relatively safe image.

To our knowledge, this novel approach is the first to collect and analyze data on all deaths for which toxicology reports were available. It is among the original pharmacoepidemiologic tools implemented by the French Addictovigilance Network^32^ and developed in partnership with the Compagnie Nationale des Biologistes et Analystes Experts.

### Limitations

This report has several limitations, particularly given the voluntary participation of toxicology experts; therefore, data collection and description of drug-related deaths throughout France is not exhaustive. In addition, only the judicial authorities can order toxicologic investigations. Despite this, approximately 80% of France was included in our study in 2021. These limitations could be overcome by increasing the geographic area covered.

## Conclusion

This case series of 4460 drug-related deaths found that opioids were the main contributor to deaths in France, similar to most countries. However, licit methadone (used alone or in combination) was the leading cause of opioid-related deaths (ahead of heroin) during the study period. Deaths related to use cannabis, NPSs, and stimulants, including ATSs and cocaine (especially in combination), increased during the study period and should be monitored closely.

Closing gaps in access to health care and harm reduction programs and addressing the social conditions of people who use drugs represent evidence-based strategies to reduce overdose rates. A large-scale naloxone distribution program is urgently needed in France to prevent opioid overdoses, including among users of licit methadone, which remains the leading drug-related cause of death. A methadone supervision program involving community pharmacists may also substantially prevent deaths due to methadone overdose.

Regarding polydrug use, raising awareness of the risks among people who use opioids or cocaine in combination with other drugs is an important strand of overdose prevention programs. The DRAMES register is continuing to collect data on drug-related deaths in France and the annual reports are available online on the National Agency for the Safety of Medicines and Health Products website.^33^

## Notes

Supplement 1. **eTable.** The homogeneity of the incidence of each drug category through time **eFigure.** Proportion of drug related-deaths involving a single (1.0) or a predominant drug (1.1) and multiple co-dominant drugs (1.2 or 1.3), by class of drug, in France, 2011-2021

Supplement 2. **Nonauthor Collaborators. **Compagnie Nationale des Biologistes et Analystes Experts (CNBAE) and the French Addictovigilance Network (FAN)

Supplement 3.
Data Sharing Statement
