# Supplementary material for: Trends in Fatal Poisoning Among Drug Users in France From 2011 to 2021: An Analysis of the DRAMES Register
Source: JAMA Netw Open. 2023 Aug 30;6(8):e2331398. doi: 10.1001/jamanetworkopen.2023.31398 (PMC10469283; doi:10.1001/jamanetworkopen.2023.31398)
Supplement: Supplement 1. — eTable. The homogeneity of the incidence of each drug category through time eFigure. Proportion of drug related-deaths involving a single (1.0) or a predominant drug (1.1) and multiple co-dominant drugs (1.2 or 1.3), by class of drug, in France, 2011-2021 [file jamanetwopen-e2331398-s001.pdf]

Supplemental Online Content

Revol B, Willeman T, Manceau M, et al; for the *Compagnie Nationale des Biologistes et Analystes Experts* (CNBAE) and the French Addictovigilance Network (FAN). Trends in fatal poisoning among drug users in France from 2011 to 2021. *JAMA Netw Open.* 2023;6(8):e2331398. doi:10.1001/jamanetworkopen.2023.31398

eTable. The homogeneity of the incidence of each drug category through time
eFigure. Proportion of drug related-deaths involving a single (1.0) or a predominant drug (1.1) and multiple co-dominant drugs (1.2 or 1.3), by class of drug, in France, 2011-2021

This supplemental material has been provided by the authors to give readers additional information about their work.

© 2023 Revol B, et al. *JAMA Netw Open.*

eTable. The homogeneity of the incidence of each drug category through time

Drug classes	2011	2012	2013	2014	2015	2016	2017	2018	2019	2020	2021	p value
Buprenorphine	30 / 185	30 / 201	24 / 182	16 / 152	20 / 206	24 / 238	22 / 268	17 / 301	25 / 335	22 / 346	30 / 405	**0.0023**
Cannabis	6 / 209	10 / 221	21 / 185	16 / 152	26 / 200	22 / 240	23 / 267	17 / 301	27 / 333	22 / 346	32 / 403	**0.0116**
Cocaine	10 / 205	18 / 213	13 / 193	9 / 159	16 / 210	28 / 234	41 / 249	45 / 273	44 / 316	35 / 333	85 / 350	**6.55e-10**
Heroin	34 / 181	28 / 203	39 / 167	38 / 130	65 / 161	68 / 194	65 / 225	86 / 232	103 / 257	93 / 275	95 / 340	**1.93e-05**
Methadone	96 / 119	112 / 119	80 / 126	77 / 91	66 / 160	87 / 175	105 / 185	106 / 212	116 / 244	150 / 218	156 / 279	**2.11e-05**
Other licit opioids	26 / 189	18 / 213	17 / 189	8 / 160	12 / 214	15 / 247	15 / 275	18 / 300	17 / 343	21 / 347	12 / 423	**0.00164**
Others	13 / 202	15 / 216	12 / 194	4 / 164	21 / 205	18 / 244	19 / 271	29 / 289	28 / 332	25 / 343	25 / 410	**0.286**

The homogeneity of the incidence of each drug category through time was assessed by means of chi-squared tests applied to each line of the table. Considering an overall 2.5% type-I error and a Bonferroni correction for the multiplicity of tests (~10), we conclude that there is significant evidence for heterogeneity through time when the p-value is less than 0.0025.

© 2023 Revol B, et al. *JAMA Netw Open.*

eFigure. Proportion of drug related-deaths involving a single (1.0) or a predominant drug (1.1) and multiple co-dominant drugs (1.2 or 1.3), by class of drug, in France, 2011-2021

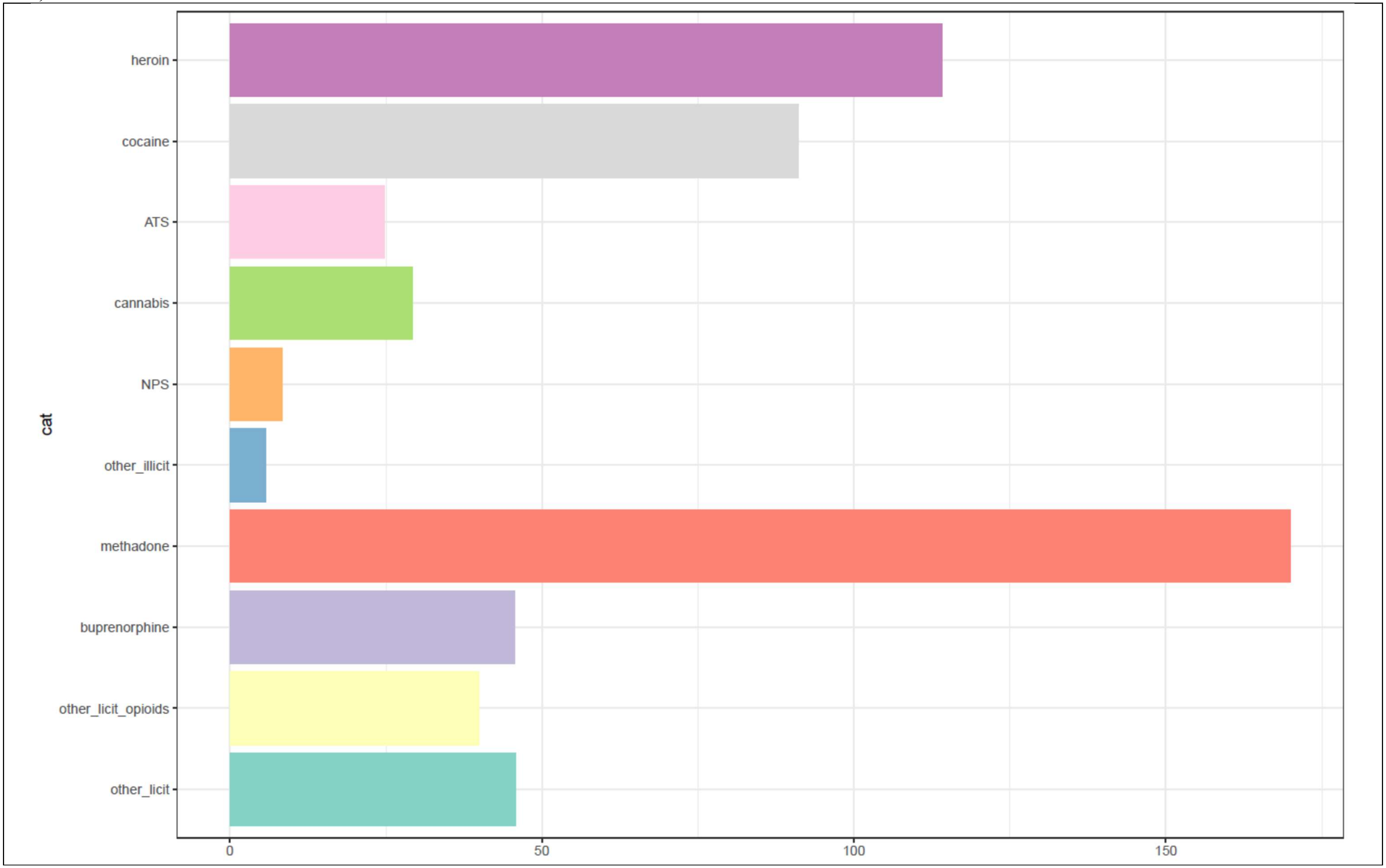

© 2023 Revol B, et al. *JAMA Netw Open.*
