# Supplementary material for: Trends in Fatal Poisoning Among Drug Users in France From 2011 to 2021: An Analysis of the DRAMES Register
Source: JAMA Netw Open. 2023 Aug 30;6(8):e2331398. doi: 10.1001/jamanetworkopen.2023.31398 (PMC10469283; doi:10.1001/jamanetworkopen.2023.31398)
Supplement: Supplement 2. — Nonauthor Collaborators. Compagnie Nationale des Biologistes et Analystes Experts (CNBAE) and the French Addictovigilance Network (FAN) [file jamanetwopen-e2331398-s002.pdf]

*Group Name: *Compagnie Nationale des Biologistes et Analystes Experts* (CNBAE) and the French Addictovigilance Network (FAN)							
***First Name and Middle Initial(s)**	***Last Name**	**Suffix (eg, Jr, III)**	Academic Degrees	Institution	Location (city, state/province, country)	Role or Contribution, eg, chair, principal investigator	Group (if more than 1 Group listed in the byline) and/or Subgroup (eg, Steering Committee)
Chadi	Abbara		PharmD, PhD	Department of Pharmacology, Toxicology and Pharmacovigilance, Angers University Hospital	Angers, France	Investigator	CNBAE
Delphine	Allorge		PharmD, PhD	Toxicology Unit, Lille University Hospital	Lille, France	Investigator	CNBAE
Jean-Claude	Alvarez		PharmD, PhD	Department of Pharmacology and Toxicology, Raymond Poincaré Hospital	Garches, France	Investigator	CNBAE
Alice	Ameline		PhD	Institut de Médecine Légale de Strasbourg	Strasbourg, France	Investigator	CNBAE
Aurélie	Aquizerate		PharmD	CEIP-Addictovigilance Nantes	Nantes, France	Investigator	FAN
Anne	Barret		PharmD	Forensic Laboratory, University Grenoble Alpes	Grenoble, France	Investigator	CNBAE
Emilie	Berland		Engineer	Service national de police scientifique - Laboratoire de Marseille	Marseille, France	Investigator	CNBAE
Célian	Bertin		MD, PhD	CEIP-Addictovigilance Clermont-Ferrand	Clermont-Ferrand, France	Investigator	FAN
Thierry	Besnard		PharmD	Laboratoire de Toxicologie et de Bioanalyse	Narbonne, France	Investigator	CNBAE
Fabien	Bevalot		PharmD, PhD	Laboratory LAT LUMTOX	Lyon, France	Investigator	CNBAE
Camille	Billet-Chatenay		PharmD	Laboratory LAT LUMTOX	Lyon, France	Investigator	CNBAE
Emilie	Bouquet		MD	CEIP-Addictovigilance Poitiers	Poitiers, France	Investigator	FAN
Joanna	Bourgine		PharmD, PhD	Department of Pharmacology, Caen University Hospital	Caen, France	Investigator	CNBAE
Bertrand	Brunet		PharmD, PhD	Biology-Pharmacy-Public Health Department,University Hospital of Poitiers	Poitiers, France	Investigator	CNBAE
Anne-Sylvie	Caous		PharmD	CEIP-Addictovigilance Lille	Lille, France	Investigator	FAN
Alexandre	Cesbron		PharmD	Department of Pharmacology, Caen University Hospital	Caen, France	Investigator	CNBAE
Leila	Chaouachi		PharmD	CEIP-Addictovigilance Paris	Paris, France	Investigator	FAN
Cécile	Chevallier		PharmD	CEIP-Addictovigilance Lyon	Lyon, France	Investigator	FAN
Marjorie	Cheze		PharmD, PhD	Laboratory Toxlab	Paris, France	Investigator	CNBAE
Antony	Citterio-Quentin		PharmD, PhD	Biochemistry and Pharmaco-toxicology Laboratory, Lyon Sud Hospital, University Hospital of Lyon	Lyon, France	Investigator	CNBAE
Philippe	Collon-Fabie		Engineer	Laboratoire de Police Scientifique	Toulouse, France	Investigator	CNBAE
Eric	Dailly		PharmD, PhD	Centre Hospitalo-Universitaire de Nantes, Service de Pharmacologie Clinique	Nantes, France	Investigator	CNBAE
Amélie	Daveluy		PharmD	CEIP-Addictovigilance Bordeaux	Bordeaux, France	Investigator	FAN
Grégory	Deffontaine		Engineer	Institut National de Police Scientifique (INPS)	Paris, France	Investigator	CNBAE
Martine	Delage		PharmD	Department of Biochemistry, Centre de Ressources Biologiques, Lapeyronie Hospital	Montpellier, France	Investigator	CNBAE
Xavier	Delavenne		PharmD, PhD	Laboratoire de Pharmacologie - Toxicologie, CHU de Saint-Etienne	St Etienne, France	Investigator	CNBAE
Florence	Descamps		Engineer	Labo Police Scientifique, LPS de Lille, France	Lille, France	Investigator	CNBAE
Juliette	Descoeur		PharmD, PhD	Department of Clinical Pharmacology, University Hospital of Montpellier	Montpellier, France	Investigator	CNBAE
Guillaume	Deslandes		PharmD	Centre Hospitalo-Universitaire de Nantes, Service de Pharmacologie Clinique	Nantes, France	Investigator	CNBAE
Marc	Deveaux		PharmD, PhD	Laboratory Toxlab	Paris, France	Investigator	CNBAE
Bernadette	Devos		Engineer	Institut National de Police Scientifique (INPS)	Paris, France	Investigator	CNBAE
Christophe	Doche		PharmD	Laboratoire de Toxicologie, Groupe Hospitalier du Havre	Le Havre, France	Investigator	CNBAE
Céline	Eiden		PharmD, PhD	CEIP-Addictovigilance Montpellier	Montpellier, France	Investigator	FAN
Aurélie	Fouley		PharmD	Department of Pharmacology, EA 3801, SFR CAP-Santé, Reims University Hospital	Reims, France	Investigator	CNBAE
Yvan	Gaillard		PharmD, PhD	Laboratory LAT LUMTOX	La Voulte-sur-Rhône, France	Investigator	CNBAE
Nicolas	Gambier		PharmD, PhD	Centre Hospitalier Régional Universitaire de Nancy (CHRU Nancy), Service de Pharmacologie Clinique et Toxi	Nancy, France	Investigator	CNBAE
Catherine	Ganière		PharmD	Centre Hospitalo-Universitaire de Nantes, Service de Pharmacologie Clinique	Nantes, France	Investigator	CNBAE
Jean-Pierre	Goullé		PharmD, PhD	Laboratoire de Toxicologie, Groupe Hospitalier du Havre	Le Havre, France	Investigator	CNBAE
Pascal	Guerard		MD, PhD	Laboratoire de Pharmacologie et Toxicologie , CHU de Dijon	Dijon, France	Investigator	CNBAE
Guillaume	Hoizey		PharmD, PhD	Laboratory Toxlab	Paris, France	Investigator	CNBAE
Luc	Humbert		Engineer	Toxicology Unit, Lille University Hospital	Lille, France	Investigator	CNBAE
Laurent	Imbert		PharmD	Laboratoire de Toxicologie, Groupe Hospitalier du Havre	Le Havre, France	Investigator	CNBAE
Marie-France	Kergueris		PharmD	Centre Hospitalo-Universitaire de Nantes, Service de Pharmacologie Clinique	Nantes, France	Investigator	CNBAE

*First Name and Middle Initial(s)	*Last Name	Suffix (eg, Jr, III)	Academic Degrees	Institution	Location (city, state/province, country)	Role or Contribution, eg, chair, principal investigator	Group (if more than 1 Group listed in the byline) and/or Subgroup (eg, Steering Committee)
Pascal	Kintz		PharmD, PhD	Institut de Médecine Légale de Strasbourg	Strasbourg, France	Investigator	CNBAE
Florian	Klinzig		PharmD	Laboratoire de Police Scientifique	Lille, France	Investigator	CNBAE
Laurence	Labat-Deveaux		PharmD, PhD	Laboratory of Toxicology, Federation of Toxicology, Lariboisière Hospital, Assistance Publique-Hôpitaux de Pa	Paris, France	Investigator	CNBAE
Bruno	Lacarelle		PharmD, PhD	Laboratory of Pharmacokinetics and Toxicology, La Timone University Hospital	Marseille, France	Investigator	CNBAE
Christian	Lacroix		PharmD	Laboratoire de Toxicologie, Groupe Hospitalier du Havre	Le Havre, France	Investigator	CNBAE
Denis	Lamiable		PharmD	Department of Pharmacology, EA 3801, SFR CAP-Santé, Reims University Hospital	Reims, France	Investigator	CNBAE
Michel	Lavit		PharmD	Pharmacokinetics and Toxicology Laboratory, Toulouse University Hospital	Toulouse, France	Investigator	CNBAE
Reynald	Le Boisselier		PharmD	CEIP-Addictovigilance Caen	Caen, France	Investigator	FAN
Anne	Le Bouil		PharmD	Pharmacology Department - Laboratory, Angers University Hospital	Angers, France	Investigator	CNBAE
Catherine	Le Meur		PharmD	Laboratory LAT LUMTOX	Lyon, France	Investigator	CNBAE
Sandrine	Lefeuvre		PharmD, PhD	Biology-Pharmacy-Public Health Department,University Hospital of Poitiers	Poitiers, France	Investigator	CNBAE
Bénédicte	Lelièvre		PharmD, PhD	Pharmacology Department - Laboratory, Angers University Hospital	Angers, France	Investigator	CNBAE
Véronique	Lelong-Boulouard		PharmD	Department of Pharmacology, Caen University Hospital	Caen, France	Investigator	CNBAE
Anne-Sophie	Lemaire-Hurtel		PharmD, PhD	Laboratory of Pharmacology and Toxicology, Department of Clinical Pharmacology, Amiens University Hospit	Amiens, France	Investigator	CNBAE
Juliana	Lima-Tournebize		PharmD, PhD	CEIP-Addictovigilance Nancy	Nancy, France	Investigator	FAN
Magalie	Loilier		PharmD	Department of Pharmacology, Caen University Hospital	Caen, France	Investigator	CNBAE
Vincent	Lopez		MD	Institut médico-légal, CHU Gabriel-Montpied	Clermont-Ferrand, France	Investigator	CNBAE
Claire	Martin-Molins		Engineer	Institut National de Police Scientifique (INPS)	Paris, France	Investigator	CNBAE
Hélène	Marty		PharmD, PhD	Department of Pharmacology, EA 3801, SFR CAP-Santé, Reims University Hospital	Reims, France	Investigator	CNBAE
Olivier	Mathieu		PharmD, PhD	Department of Clinical Pharmacology, University Hospital of Montpellier	Montpellier, France	Investigator	CNBAE
Jean-Claude	Mathieu-Daudé		MD	Laboratoire de Toxicologie, Département de Pharmacologie médicale et Toxicologie, Hôpital Lapeyronie, CH	Montpellier, France	Investigator	CNBAE
Yves	Mauras		PharmD, PhD	Pharmacology Department - Laboratory, Angers University Hospital	Angers, France	Investigator	CNBAE
Nathalie	Milan		Engineer	Institut National de Police Scientifique (INPS)	Paris, France	Investigator	CNBAE
Aurélie	Moal		Engineer	Institut National de Police Scientifique (INPS)	Paris, France	Investigator	CNBAE
Isabelle	Morel		PharmD, PhD	Centre Hospitalier Universitaire de Rennes, Laboratoire de Toxicologie Biologique et Medico-Légale, CHU Po	Rennes, France	Investigator	CNBAE
Patrick	Mura		PharmD, PhD	Biology-Pharmacy-Public Health Department,University Hospital of Poitiers	Poitiers, France	Investigator	CNBAE
Anne-Laure	Pelissier-Alicot		MD, PhD	Service de Médecine Légale, Aix-Marseille Université	Marseille, France	Investigator	CNBAE
Gilbert	Pépin		PharmD, PhD	Laboratory Toxlab	Paris, France	Investigator	CNBAE
Martine	Perrin		PharmD	Institut de Recherche Criminelle de la Gendarmerie Nationale (IRCGN)	Rosny-sous-bois, France	Investigator	CNBAE
Anne	Peyre		Engineer	Service national de police scientifique - Laboratoire de Marseille	Marseille, France	Investigator	CNBAE
Alain	Pineau		PharmD, PhD	Centre Hospitalo-Universitaire de Nantes, Service de Pharmacologie Clinique	Nantes, France	Investigator	CNBAE
Rop	Pok		PharmD	Service national de police scientifique - Laboratoire de Marseille	Marseille, France	Investigator	CNBAE
Catherine	Ragoucy-Sengler		PharmD	Laboratory Toxlab	Paris, France	Investigator	CNBAE
Raphaël	Rayer		Engineer	Laboratoire de Police Scientifique	Lille, France	Investigator	CNBAE
Emilie	Roman		PharmD	Laboratoire de biochimie-toxicologie, Centre hospitalier régional Metz-Thionville	Thionville, France	Investigator	CNBAE
Carine	Roussel		Engineer	Laboratoire de Police Scientifique	Toulouse, France	Investigator	CNBAE
Anne	Roussin		PharmD, PhD	CEIP-Addictovigilance Toulouse	Toulouse, France	Investigator	FAN
Sandrine	Sabini		PharmD	Institut de Recherche Criminelle de la Gendarmerie Nationale (IRCGN)	Rosny-sous-Bois, France	Investigator	CNBAE
Elodie	Saussereau		PharmD	Laboratoire de Toxicologie, Groupe Hospitalier du Havre	Le Havre, France	Investigator	CNBAE
Julien	Scala-Bertola		PharmD, PhD	Centre Hospitalier Régional Universitaire de Nancy (CHRU Nancy), Service de Pharmacologie Clinique et Toxi	Nancy, France	Investigator	CNBAE
Pauline	Sibille		Engineer	Institut National de Police Scientifique (INPS)	Paris, France	Investigator	CNBAE
Michel	Spadari		MD	CEIP-Addictovigilance Marseille	Marseille, France	Investigator	FAN
Karine	Titier		PharmD, PhD	CHU de Bordeaux, Laboratoire de Pharmacologie et Toxicologie	Bordeaux, France	Investigator	CNBAE
Alain	Turcant		PharmD, PhD	Pharmacology Department - Laboratory, Angers University Hospital	Angers, France	Investigator	CNBAE

*First Name and Middle Initial(s)	*Last Name	Suffix (eg, Jr, III)	Academic Degrees	Institution	Location (city, state/province, country)	Role or Contribution, eg, chair, principal investigator	Group (if more than 1 Group listed in the byline) and/or Subgroup (eg, Steering Committee)
Pierrick	Vacher		MD	Laboratoire de Pharmacologie - Toxicologie, CHU de Saint-Etienne	Saint Etienne, France	Investigator	CNBAE
Nicolas	Venisse		PharmD, PhD	Biology-Pharmacy-Public Health Department,University Hospital of Poitiers	Poitiers, France	Investigator	CNBAE
Ophélie	Vieira		Engineer	Laboratoire de Police Scientifique	Paris, France	Investigator	CNBAE
Pascale	Visinoni		Engineer	Laboratoire de Police Scientifique	Toulouse, France	Investigator	CNBAE
