# Supplementary material for: Trends in Fatal Poisoning Among Drug Users in France From 2011 to 2021: An Analysis of the DRAMES Register
Source: JAMA Netw Open. 2023 Aug 30;6(8):e2331398. doi: 10.1001/jamanetworkopen.2023.31398 (PMC10469283; doi:10.1001/jamanetworkopen.2023.31398)
Supplement: Supplement 3. — Data Sharing Statement [file jamanetwopen-e2331398-s003.pdf]

Data Sharing Statement

Data
Data available: No

Additional Information
Explanation for why data not available: Annual reports are already available online on the ANSM website: https://ansm.sante.fr/page/resultats-denquetes-pharmacodependance-addictovigilance
